# Dichlorido[2-(3,5-dimethyl-1*H*-pyrazol-1-yl-κ*N* ^2^)quinoline-κ*N*]zinc

**DOI:** 10.1107/S1600536812014390

**Published:** 2012-04-13

**Authors:** Muhd. Hidayat bin Najib, Ai Ling Tan, David J. Young, Seik Weng Ng, Edward R. T. Tiekink

**Affiliations:** a Faculty of Science, Universiti Brunei Darussalam, Jalan Tungku Link BE 1410, Negara Brunei Darussalam; b Department of Chemistry, University of Malaya, 50603 Kuala Lumpur, Malaysia; c Chemistry Department, Faculty of Science, King Abdulaziz University, PO Box 80203 Jeddah, Saudi Arabia

## Abstract

The Zn^II^ atom in the title compound, [ZnCl_2_(C_14_H_13_N_3_)], is coordinated by a Cl_2_N_2_ donor set defined by quinoline and pyrazole N atoms of the chelating ligand and two Cl atoms. Distortions from the ideal tetra­hedral geometry relate to the restricted bite angle of the chelating ligand [N—Zn—N = 78.54 (12)°]. In the crystal, mol­ecules are connected into a three-dimensional structure by C—H⋯Cl inter­actions, involving both Cl atoms, and π–π inter­actions that occur between the pyrazole ring and each of the pyridine and benzene rings of the quinoline residue [inter­centroid distances = 3.655 (2) and 3.676 (2) Å].

## Related literature

For background to luminescent coordination complexes, see: Bai *et al.* (2012 ▶); Chou *et al.*, (2011 ▶); Hu *et al.* (2011 ▶); Wang (2001 ▶). For the synthesis, see: Savel’eva *et al.* (2009 ▶); Scott *et al.* (1952 ▶).
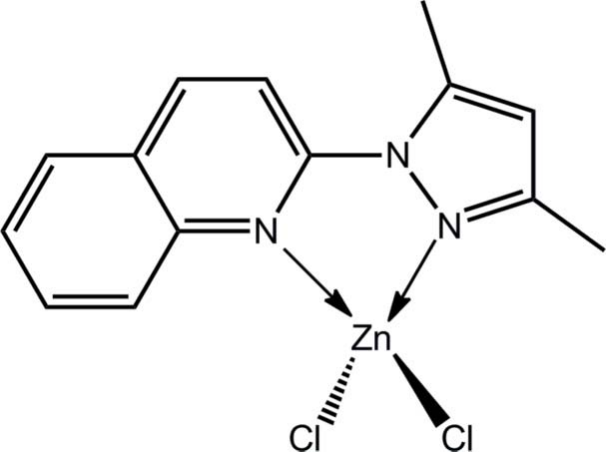


## Experimental

### 

#### Crystal data

[ZnCl_2_(C_14_H_13_N_3_)]
*M* *_r_* = 359.54
Monoclinic, 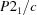

*a* = 14.3353 (10) Å
*b* = 8.7683 (5) Å
*c* = 11.9839 (8) Å
β = 102.181 (7)°
*V* = 1472.42 (17) Å^3^
*Z* = 4
Mo *K*α radiation
μ = 2.02 mm^−1^
*T* = 100 K
0.25 × 0.20 × 0.02 mm

#### Data collection

Agilent SuperNova Dual diffractometer with an Atlas detector
Absorption correction: multi-scan (*CrysAlis PRO*; Agilent, 2010 ▶) *T*_min_ = 0.597, *T*_max_ = 1.000
6070 measured reflections
3370 independent reflections
2438 reflections with *I* > 2σ(*I*)
*R*_int_ = 0.047

#### Refinement

*R*[*F* ^2^ > 2σ(*F* ^2^)] = 0.050
*wR*(*F* ^2^) = 0.124
*S* = 1.02
3370 reflections
183 parameters
H-atom parameters constrained
Δρ_max_ = 1.04 e Å^−3^
Δρ_min_ = −0.66 e Å^−3^

### 

Data collection: *CrysAlis PRO* (Agilent, 2010 ▶); cell refinement: *CrysAlis PRO*; data reduction: *CrysAlis PRO*; program(s) used to solve structure: *SHELXS97* (Sheldrick, 2008 ▶); program(s) used to refine structure: *SHELXL97* (Sheldrick, 2008 ▶); molecular graphics: *ORTEP-3* (Farrugia, 1997 ▶) and *DIAMOND* (Brandenburg, 2006 ▶); software used to prepare material for publication: *publCIF* (Westrip, 2010 ▶).

## Supplementary Material

Crystal structure: contains datablock(s) global, I. DOI: 10.1107/S1600536812014390/hg5206sup1.cif

Structure factors: contains datablock(s) I. DOI: 10.1107/S1600536812014390/hg5206Isup2.hkl

Additional supplementary materials:  crystallographic information; 3D view; checkCIF report

## Figures and Tables

**Table 1:** Selected bond lengths (Å)

Zn—N1	2.021 (3)
Zn—N3	2.072 (3)
Zn—Cl1	2.2099 (11)
Zn—Cl2	2.2076 (11)

**Table 2:** Hydrogen-bond geometry (Å, °)

*D*—H⋯*A*	*D*—H	H⋯*A*	*D*⋯*A*	*D*—H⋯*A*
C1—H1*A*⋯Cl1^i^	0.98	2.81	3.680 (4)	148
C12—H12*A*⋯Cl2^ii^	0.95	2.82	3.579 (4)	138

Symmetry codes: (i) 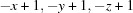; (ii) 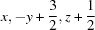.

**Fig. 1.**
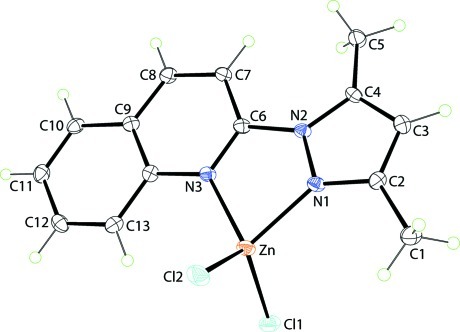
The molecular structure of (I) showing the atom-labelling scheme and displacement ellipsoids at the 50% probability level.

**Fig. 2.**
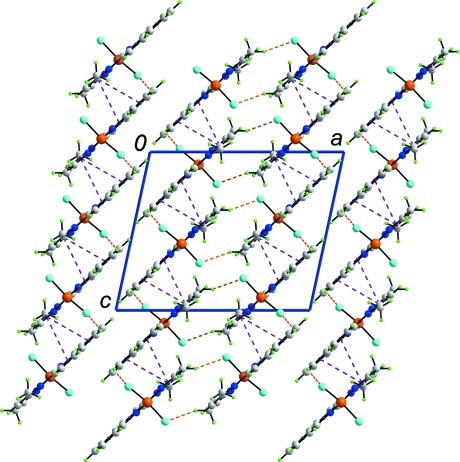
A view of the unit-cell contents of (I) in projection down the b axis. The C—H···Cl and π—π interactions are shown as orange and purple dashed lines, respectively.

## supplementary crystallographic information

### Comment

Luminescent coordination complexes are used as emitting materials in light-emitting devices (Chou *et al.*, 2011) and it is known that many Zn^II^ nitrogen donor complexes are brightly luminescent in the blue region of the spectrum (Wang, 2001). The title compound (I) was prepared as part of our on-going quest for luminescent organometallic and coordination complexes with improved quantum efficiencies and colour purity (Bai *et al.*, 2012; Hu *et al.*, 2011;).

The Zn^II^ atom in (I), Fig. 1, is chelated by quinolinyl- and pyrazolyl-N atoms and two chloride atoms, Table 1. The resulting Cl_2_N_2_ donor set defines a tetrahedron with a significant distortion apparent owing to the restricted bite angle of the chelating ligand, *i.e*. N1—Zn—N3 78.54 (12)°; the remaining angles lie in the range 111.52 (9)°, for N1—Zn—Cl1, to 115.73 (9)°, for N1—Zn—Cl2. The five-membered chelate ring is essentially planar with a r.m.s. deviation = 0.035 Å with maximum deviations of 0.034 (3) and -0.029 (3) Å for the N2 and N1 atoms, respectively. A small twist is apparent in the bidentate ligand with the dihedral angle between the quinolinyl and pyrazolyl rings being 6.84 (17)°. The overall coordination geometry found for (I) matches that seen in the species carrying an additional methyl group in the 4-position of the quinolinyl residue (Savel'eva *et al.*, 2009).

In the crystal packing, Fig. 2, molecules are connected into a three-dimensional architecture by C—H···Cl interactions, Table 1, involving both chloride atoms, and π—π interactions between the pyrazoyl ring and each of the pyridyl and benzene rings of the quinolinyl residue [inter-centroid distances = 3.655 (2) and 3.676 (2) Å, respectively; angles of inclination = 3.2 (2) and 3.5 (2)°, respectively; for symmetry operation: *x*, 1/2 - *y*, -1/2 + *z*].

### Experimental

The title compound was prepared by modification of literature procedures (Savel'eva *et al.*, 2009; Scott *et al.* 1952). 3,5-Dimethyl-1-(2'-quinolyl)pyrazole (0.080 g) in a mixture of EtOH (4 ml) and CH_2_Cl_2_ (2 ml) was added to a solution of ZnCl_2_ (0.061 g) in EtOH (8 ml). A white precipitate formed and was collected by filtration after 1 h, washed with EtOH and air-dried. The crude product was recrystallized from its CH_2_Cl_2_/hexane solution. Yield 0.054 g (42%). *M*. pt: 607–608 K. IR ν/cm^-1^: 1619, 1596, 1579, 1559, 1511, 1479, 1439, 1386, 1380, 1346, 1319, 1142, 1090, 994, 983, 825, 783, 760.

### Refinement

Carbon-bound H-atoms were placed in calculated positions [C—H = 0.95–0.98 Å, *U*_iso_(H) = 1.2–1.5*U*_eq_(C)] and were included in the refinement in the riding model approximation. The maximum and minimum residual electron density peaks of 1.04 and 0.66 e Å^-3^, respectively, were located 1.00 Å and 0.95 Å from the Zn atom.

### Crystal data

**Table:**

[ZnCl_2_(C_14_H_13_N_3_)]	*F*(000) = 728
*M**_r_* = 359.54	*D*_x_ = 1.622 Mg m^−^^3^
Monoclinic, *P*2_1_/*c*	Mo *K*α radiation, λ = 0.71073 Å
Hall symbol: -P 2ybc	Cell parameters from 1666 reflections
*a* = 14.3353 (10) Å	θ = 2.5–27.5°
*b* = 8.7683 (5) Å	µ = 2.02 mm^−^^1^
*c* = 11.9839 (8) Å	*T* = 100 K
β = 102.181 (7)°	Plate, colourless
*V* = 1472.42 (17) Å^3^	0.25 × 0.20 × 0.02 mm
*Z* = 4	

### Data collection

**Table:**

Agilent SuperNova Dual diffractometer with an Atlas detector	3370 independent reflections
Radiation source: SuperNova (Mo) X-ray Source	2438 reflections with *I* > 2σ(*I*)
Mirror monochromator	*R*_int_ = 0.047
Detector resolution: 10.4041 pixels mm^-1^	θ_max_ = 27.6°, θ_min_ = 2.7°
ω scan	*h* = −17→18
Absorption correction: multi-scan (*CrysAlis PRO*; Agilent, 2010)	*k* = −11→8
*T*_min_ = 0.597, *T*_max_ = 1.000	*l* = −9→15
6070 measured reflections	

### Refinement

**Table:**

Refinement on *F*^2^	Primary atom site location: structure-invariant direct methods
Least-squares matrix: full	Secondary atom site location: difference Fourier map
*R*[*F*^2^ > 2σ(*F*^2^)] = 0.050	Hydrogen site location: inferred from neighbouring sites
*wR*(*F*^2^) = 0.124	H-atom parameters constrained
*S* = 1.02	*w* = 1/[σ^2^(*F*_o_^2^) + (0.0519*P*)^2^ + 0.2907*P*] where *P* = (*F*_o_^2^ + 2*F*_c_^2^)/3
3370 reflections	(Δ/σ)_max_ = 0.001
183 parameters	Δρ_max_ = 1.04 e Å^−^^3^
0 restraints	Δρ_min_ = −0.66 e Å^−^^3^

### Special details

**Table:**

Geometry. All e.s.d.'s (except the e.s.d. in the dihedral angle between two l.s. planes) are estimated using the full covariance matrix. The cell e.s.d.'s are taken into account individually in the estimation of e.s.d.'s in distances, angles and torsion angles; correlations between e.s.d.'s in cell parameters are only used when they are defined by crystal symmetry. An approximate (isotropic) treatment of cell e.s.d.'s is used for estimating e.s.d.'s involving l.s. planes.
Refinement. Refinement of *F*^2^ against ALL reflections. The weighted *R*-factor *wR* and goodness of fit *S* are based on *F*^2^, conventional *R*-factors *R* are based on *F*, with *F* set to zero for negative *F*^2^. The threshold expression of *F*^2^ > σ(*F*^2^) is used only for calculating *R*-factors(gt) *etc*. and is not relevant to the choice of reflections for refinement. *R*-factors based on *F*^2^ are statistically about twice as large as those based on *F*, and *R*- factors based on ALL data will be even larger.

### Fractional atomic coordinates and isotropic or equivalent isotropic displacement parameters (Å^2^)

**Table:**

	*x*	*y*	*z*	*U*_iso_*/*U*_eq_	
Zn	0.26453 (3)	0.53715 (5)	0.58123 (4)	0.02140 (16)	
Cl1	0.37804 (7)	0.65835 (11)	0.70222 (9)	0.0296 (3)	
Cl2	0.14937 (7)	0.67975 (11)	0.48204 (9)	0.0300 (3)	
N1	0.3193 (2)	0.3781 (3)	0.4912 (3)	0.0185 (7)	
N3	0.2198 (2)	0.3372 (3)	0.6458 (3)	0.0179 (7)	
C1	0.4079 (3)	0.5145 (4)	0.3689 (4)	0.0255 (9)	
H1A	0.4758	0.5071	0.3671	0.038*	
H1B	0.3979	0.6015	0.4163	0.038*	
H1C	0.3706	0.5289	0.2911	0.038*	
C2	0.3767 (3)	0.3710 (4)	0.4180 (3)	0.0194 (8)	
C3	0.4000 (3)	0.2193 (4)	0.4009 (3)	0.0200 (8)	
H3	0.4400	0.1841	0.3524	0.024*	
C4	0.3546 (2)	0.1306 (4)	0.4674 (3)	0.0186 (8)	
C5	0.3603 (3)	−0.0381 (4)	0.4822 (4)	0.0249 (9)	
H5A	0.3798	−0.0626	0.5636	0.037*	
H5B	0.4073	−0.0793	0.4415	0.037*	
H5C	0.2977	−0.0833	0.4513	0.037*	
C6	0.2458 (2)	0.2097 (4)	0.6031 (3)	0.0174 (8)	
C7	0.2184 (3)	0.0628 (4)	0.6327 (3)	0.0189 (8)	
H7A	0.2373	−0.0265	0.5984	0.023*	
C8	0.1632 (3)	0.0549 (4)	0.7129 (3)	0.0207 (8)	
H8A	0.1442	−0.0421	0.7358	0.025*	
C9	0.1340 (2)	0.1877 (4)	0.7623 (3)	0.0186 (8)	
C10	0.0765 (2)	0.1852 (4)	0.8457 (3)	0.0216 (8)	
H10A	0.0563	0.0905	0.8710	0.026*	
C11	0.0505 (3)	0.3181 (5)	0.8891 (3)	0.0243 (9)	
H11A	0.0119	0.3161	0.9446	0.029*	
C12	0.0805 (3)	0.4586 (4)	0.8521 (3)	0.0229 (9)	
H12A	0.0617	0.5506	0.8830	0.027*	
C13	0.1360 (3)	0.4656 (4)	0.7729 (3)	0.0222 (8)	
H13A	0.1565	0.5614	0.7499	0.027*	
C14	0.1628 (2)	0.3301 (4)	0.7257 (3)	0.0187 (8)	
N2	0.3048 (2)	0.2299 (3)	0.5218 (3)	0.0173 (7)	

### Atomic displacement parameters (Å^2^)

**Table:**

	*U*^11^	*U*^22^	*U*^33^	*U*^12^	*U*^13^	*U*^23^
Zn	0.0306 (3)	0.0144 (2)	0.0210 (3)	−0.00055 (19)	0.00946 (19)	0.00027 (19)
Cl1	0.0423 (6)	0.0242 (5)	0.0237 (6)	−0.0104 (4)	0.0104 (4)	−0.0047 (4)
Cl2	0.0364 (6)	0.0193 (5)	0.0355 (6)	0.0071 (4)	0.0102 (5)	0.0038 (4)
N1	0.0250 (16)	0.0152 (16)	0.0151 (16)	−0.0005 (13)	0.0042 (13)	−0.0004 (13)
N3	0.0248 (16)	0.0180 (16)	0.0116 (16)	−0.0026 (13)	0.0054 (13)	−0.0017 (13)
C1	0.031 (2)	0.024 (2)	0.021 (2)	−0.0040 (18)	0.0050 (17)	0.0009 (17)
C2	0.0212 (18)	0.023 (2)	0.0139 (19)	−0.0034 (16)	0.0029 (15)	0.0013 (16)
C3	0.0205 (18)	0.024 (2)	0.0145 (19)	0.0024 (16)	0.0024 (14)	0.0005 (16)
C4	0.0213 (18)	0.0184 (19)	0.0154 (19)	0.0020 (16)	0.0025 (15)	−0.0039 (16)
C5	0.033 (2)	0.017 (2)	0.028 (2)	0.0027 (17)	0.0137 (18)	0.0029 (17)
C6	0.0211 (19)	0.0185 (19)	0.0113 (19)	−0.0014 (16)	0.0004 (14)	−0.0012 (15)
C7	0.0243 (19)	0.0157 (18)	0.0168 (19)	−0.0003 (16)	0.0047 (15)	−0.0017 (15)
C8	0.027 (2)	0.0168 (19)	0.019 (2)	−0.0016 (16)	0.0064 (16)	0.0047 (16)
C9	0.0199 (18)	0.0198 (19)	0.0149 (19)	−0.0009 (16)	0.0011 (14)	0.0004 (16)
C10	0.024 (2)	0.023 (2)	0.018 (2)	−0.0061 (16)	0.0044 (16)	0.0004 (17)
C11	0.024 (2)	0.033 (2)	0.018 (2)	−0.0044 (18)	0.0078 (16)	−0.0030 (18)
C12	0.0244 (19)	0.025 (2)	0.019 (2)	0.0049 (17)	0.0024 (16)	−0.0033 (17)
C13	0.0230 (19)	0.023 (2)	0.020 (2)	−0.0048 (17)	0.0036 (16)	0.0005 (17)
C14	0.0191 (18)	0.0210 (19)	0.0153 (19)	0.0005 (16)	0.0020 (15)	0.0021 (16)
N2	0.0230 (16)	0.0139 (15)	0.0152 (16)	−0.0003 (13)	0.0039 (13)	0.0007 (13)

### Geometric parameters (Å, º)

**Table:**

Zn—N1	2.021 (3)	C5—H5B	0.9800
Zn—N3	2.072 (3)	C5—H5C	0.9800
Zn—Cl1	2.2099 (11)	C6—C7	1.414 (5)
Zn—Cl2	2.2076 (11)	C6—N2	1.429 (5)
N1—C2	1.325 (5)	C7—C8	1.370 (5)
N1—N2	1.378 (4)	C7—H7A	0.9500
N3—C6	1.316 (5)	C8—C9	1.409 (5)
N3—C14	1.385 (5)	C8—H8A	0.9500
C1—C2	1.497 (5)	C9—C14	1.414 (5)
C1—H1A	0.9800	C9—C10	1.423 (5)
C1—H1B	0.9800	C10—C11	1.361 (5)
C1—H1C	0.9800	C10—H10A	0.9500
C2—C3	1.397 (5)	C11—C12	1.407 (5)
C3—C4	1.373 (5)	C11—H11A	0.9500
C3—H3	0.9500	C12—C13	1.362 (5)
C4—N2	1.375 (4)	C12—H12A	0.9500
C4—C5	1.490 (5)	C13—C14	1.403 (5)
C5—H5A	0.9800	C13—H13A	0.9500
			
N1—Zn—N3	78.54 (12)	H5B—C5—H5C	109.5
N1—Zn—Cl2	115.73 (9)	N3—C6—C7	124.0 (3)
N3—Zn—Cl2	115.15 (9)	N3—C6—N2	114.6 (3)
N1—Zn—Cl1	111.52 (9)	C7—C6—N2	121.3 (3)
N3—Zn—Cl1	113.89 (9)	C8—C7—C6	117.0 (3)
Cl2—Zn—Cl1	116.38 (4)	C8—C7—H7A	121.5
C2—N1—N2	106.4 (3)	C6—C7—H7A	121.5
C2—N1—Zn	138.7 (3)	C7—C8—C9	121.3 (3)
N2—N1—Zn	114.3 (2)	C7—C8—H8A	119.3
C6—N3—C14	119.2 (3)	C9—C8—H8A	119.3
C6—N3—Zn	116.0 (2)	C8—C9—C14	117.9 (3)
C14—N3—Zn	124.8 (2)	C8—C9—C10	123.3 (3)
C2—C1—H1A	109.5	C14—C9—C10	118.8 (3)
C2—C1—H1B	109.5	C11—C10—C9	120.1 (4)
H1A—C1—H1B	109.5	C11—C10—H10A	119.9
C2—C1—H1C	109.5	C9—C10—H10A	119.9
H1A—C1—H1C	109.5	C10—C11—C12	120.2 (4)
H1B—C1—H1C	109.5	C10—C11—H11A	119.9
N1—C2—C3	110.0 (3)	C12—C11—H11A	119.9
N1—C2—C1	120.0 (3)	C13—C12—C11	121.4 (4)
C3—C2—C1	129.9 (3)	C13—C12—H12A	119.3
C4—C3—C2	107.3 (3)	C11—C12—H12A	119.3
C4—C3—H3	126.3	C12—C13—C14	119.5 (4)
C2—C3—H3	126.3	C12—C13—H13A	120.3
C3—C4—N2	105.9 (3)	C14—C13—H13A	120.3
C3—C4—C5	127.7 (3)	N3—C14—C13	119.5 (3)
N2—C4—C5	126.4 (3)	N3—C14—C9	120.5 (3)
C4—C5—H5A	109.5	C13—C14—C9	120.0 (3)
C4—C5—H5B	109.5	C4—N2—N1	110.4 (3)
H5A—C5—H5B	109.5	C4—N2—C6	133.3 (3)
C4—C5—H5C	109.5	N1—N2—C6	116.3 (3)
H5A—C5—H5C	109.5		
			
N3—Zn—N1—C2	−172.9 (4)	C7—C8—C9—C10	179.8 (4)
Cl2—Zn—N1—C2	74.6 (4)	C8—C9—C10—C11	−179.6 (3)
Cl1—Zn—N1—C2	−61.5 (4)	C14—C9—C10—C11	−0.2 (5)
N3—Zn—N1—N2	−3.5 (2)	C9—C10—C11—C12	−0.2 (5)
Cl2—Zn—N1—N2	−116.1 (2)	C10—C11—C12—C13	−0.2 (6)
Cl1—Zn—N1—N2	107.8 (2)	C11—C12—C13—C14	1.0 (6)
N1—Zn—N3—C6	0.5 (2)	C6—N3—C14—C13	179.0 (3)
Cl2—Zn—N3—C6	113.7 (2)	Zn—N3—C14—C13	−3.6 (5)
Cl1—Zn—N3—C6	−108.2 (2)	C6—N3—C14—C9	0.9 (5)
N1—Zn—N3—C14	−177.0 (3)	Zn—N3—C14—C9	178.3 (2)
Cl2—Zn—N3—C14	−63.8 (3)	C12—C13—C14—N3	−179.5 (3)
Cl1—Zn—N3—C14	74.3 (3)	C12—C13—C14—C9	−1.5 (5)
N2—N1—C2—C3	−0.2 (4)	C8—C9—C14—N3	−1.5 (5)
Zn—N1—C2—C3	169.6 (3)	C10—C9—C14—N3	179.1 (3)
N2—N1—C2—C1	−179.2 (3)	C8—C9—C14—C13	−179.6 (3)
Zn—N1—C2—C1	−9.3 (6)	C10—C9—C14—C13	1.1 (5)
N1—C2—C3—C4	−0.1 (4)	C3—C4—N2—N1	−0.6 (4)
C1—C2—C3—C4	178.7 (4)	C5—C4—N2—N1	177.2 (3)
C2—C3—C4—N2	0.5 (4)	C3—C4—N2—C6	−178.4 (4)
C2—C3—C4—C5	−177.3 (4)	C5—C4—N2—C6	−0.6 (6)
C14—N3—C6—C7	0.7 (5)	C2—N1—N2—C4	0.5 (4)
Zn—N3—C6—C7	−176.9 (3)	Zn—N1—N2—C4	−172.2 (2)
C14—N3—C6—N2	−179.8 (3)	C2—N1—N2—C6	178.7 (3)
Zn—N3—C6—N2	2.6 (4)	Zn—N1—N2—C6	6.0 (4)
N3—C6—C7—C8	−1.7 (5)	N3—C6—N2—C4	172.0 (3)
N2—C6—C7—C8	178.9 (3)	C7—C6—N2—C4	−8.5 (6)
C6—C7—C8—C9	1.0 (6)	N3—C6—N2—N1	−5.7 (4)
C7—C8—C9—C14	0.5 (5)	C7—C6—N2—N1	173.8 (3)

### Hydrogen-bond geometry (Å, º)

**Table:**

*D*—H···*A*	*D*—H	H···*A*	*D*···*A*	*D*—H···*A*
C1—H1*A*···Cl1^i^	0.98	2.81	3.680 (4)	148
C12—H12*A*···Cl2^ii^	0.95	2.82	3.579 (4)	138

Symmetry codes: (i) −*x*+1, −*y*+1, −*z*+1; (ii) *x*, −*y*+3/2, *z*+1/2.
